# UNI-EM: An Environment for Deep Neural Network-Based Automated Segmentation of Neuronal Electron Microscopic Images

**DOI:** 10.1038/s41598-019-55431-0

**Published:** 2019-12-19

**Authors:** Hidetoshi Urakubo, Torsten Bullmann, Yoshiyuki Kubota, Shigeyuki Oba, Shin Ishii

**Affiliations:** 1 Integrated Systems Biology Laboratory, Department of Systems Science, Graduate School of Informatics, Kyoto University, Yoshida-Honmachi 36-1, Sakyo-ku, Kyoto 606-8501 Japan; 2 Carl-Ludwig-Institute for Physiology, University Leipzig, Liebigstr. 27, 04103 Leipzig, Germany; 3 Division of Cerebral Circuitry, National Institute for Physiological Sciences, 5-1 Myodaiji-Higashiyama, Okazaki, Aichi 444-8787 Japan; 4 Department of Physiological Sciences, The Graduate University for Advanced Studies (SOKENDAI), 5-1 Myodaiji-Higashiyama, Okazaki, Aichi 444-8787 Japan

**Keywords:** Software, Neural circuits, Software

## Abstract

Recently, there has been rapid expansion in the field of micro-connectomics, which targets the three-dimensional (3D) reconstruction of neuronal networks from stacks of two-dimensional (2D) electron microscopy (EM) images. The spatial scale of the 3D reconstruction increases rapidly owing to deep convolutional neural networks (CNNs) that enable automated image segmentation. Several research teams have developed their own software pipelines for CNN-based segmentation. However, the complexity of such pipelines makes their use difficult even for computer experts and impossible for non-experts. In this study, we developed a new software program, called UNI-EM, for 2D and 3D CNN-based segmentation. UNI-EM is a software collection for CNN-based EM image segmentation, including ground truth generation, training, inference, postprocessing, proofreading, and visualization. UNI-EM incorporates a set of 2D CNNs, i.e., U-Net, ResNet, HighwayNet, and DenseNet. We further wrapped flood-filling networks (FFNs) as a representative 3D CNN-based neuron segmentation algorithm. The 2D- and 3D-CNNs are known to demonstrate state-of-the-art level segmentation performance. We then provided two example workflows: mitochondria segmentation using a 2D CNN and neuron segmentation using FFNs. By following these example workflows, users can benefit from CNN-based segmentation without possessing knowledge of Python programming or CNN frameworks.

## Introduction

In recent years, there has been a rapid expansion in the field of micro-connectomics, which targets the three-dimensional (3D) reconstruction of neuronal networks from stacks of two-dimensional (2D) electron microscopy (EM) images^1–3^. Neuroscientists have successfully reconstructed large-scale neural circuits from species, such as mice^4^, fruit flies^5^, and zebrafish^6^. Such large-scale reconstructions require neuronal boundary detection (or neuron segmentation) of large numbers of EM images, and automation is critical even for smaller-scale segmentation.

For automated neuron segmentation, studies have validated the effectiveness of deep convolutional neural networks (CNNs)^7^. In particular, U-Net, which is a type of CNN, showed the highest accuracy in a neuron segmentation contest^8^, and similar CNNs also proved effective^9–11^. Three-dimensional CNNs have also been developed for higher segmentation accuracy. Januszewski *et al*. developed a type of recursive 3D CNN called flood filling networks (FFNs)^12^, which showed the highest segmentation accuracy in a public 3D EM dataset (FIB-25)^13^ and the second highest in another public 3D EM dataset (3D segmentation of neurites in EM images, SNEMI3D)^14^. Therefore, the use of such CNNs has become critical for accurate neuron segmentation.

Most CNN source codes are publicly available; however, it is not easy to perform segmentation even with these source codes. Users are required to prepare ground truth segmentation for their own EM images first and then to conduct preprocessing tasks, such as data conversion. The preprocessing and use of CNNs often require users to learn the underlying programming language, which is generally Python. After performing CNN-based segmentation, users need to conduct postprocessing, including proofreading, annotation, and visualization. In short, CNN-based neuron segmentation, although an important task, constitutes only a portion of the entire segmentation procedure.

Advanced connectomics laboratories have developed their own software pipelines to employ CNN-based segmentation, including Rhoana^15,16^, Eyewire^17^, and the FFN segmentation pipeline^5^. The main objective of these pipelines is large-scale 3D reconstructions that are conducted by large teams including computer experts for setup and maintenance. They are too complicated to be used by smaller teams. EM segmentation is also handled by sophisticated standalone software packages, such as Reconstruct^18^, Ilastik^19^, Knossos^20^, Microscopy Image Browser^21^, and VAST lite^22^. However, most software packages only target manual segmentation^18,20,22^, and others currently do not support CNN-based segmentation^19,21^. Recently, a plug-in for the widely used ImageJ software was developed to handle CNN-based segmentation^23^. The use of this plug-in is advantageous; however, it currently provides only four types of U-Net models, and users need to launch a server on a Linux computer to train the U-Nets.

We therefore developed a unified environment for CNN-based automated segmentation of EM images (UNI-EM) for researchers with limited programming skills. UNI-EM implements several 2D CNNs^8–11^ and 3D FFNs^12^ on the widely used Tensorflow framework/Python^24^. It also includes the proofreading software Dojo^25^ as well as a series of 2D/3D filters for classic image processing. Those features enable users to follow the procedure of CNN-based segmentation, i.e., ground truth generation, training, inference, postprocessing, proofreading, and visualization. UNI-EM currently supports two major operating systems (OSs): Microsoft Windows 10 (64 bit) and Linux. We also provide Python installation-free versions of UNI-EM (Pyinstaller version). Thus, users do not need to install Python or any modules for CNN-based segmentation.

## Results

### Outline of software

**Figure 1:**
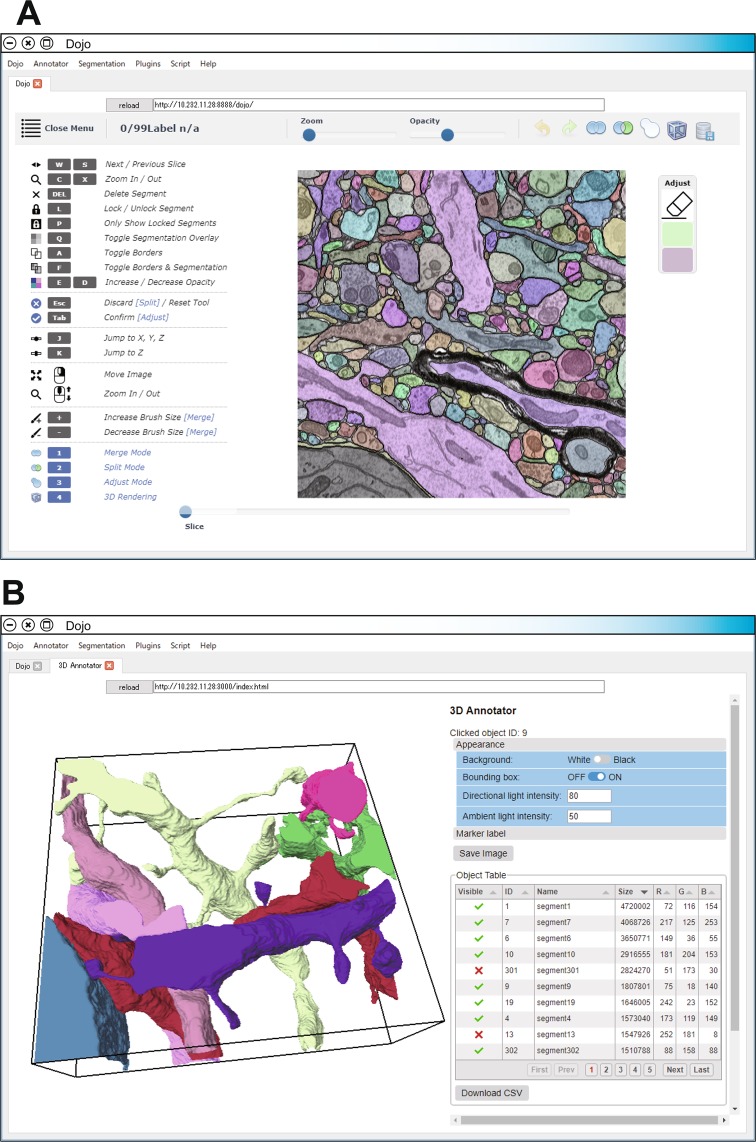
GUIs of UNI-EM. (**A**) Proofreader Dojo with extension. The GUI of Dojo was reorganized. Users can rectify mis-segmentation as well as build the ground truth using paint functions. The reorganized Dojo supports the import/export functions of EM/segmentation image stack files. (**B**) 3D annotator. A 3D viewer (left) is associated with the object tables (right) that display segmented object and marker points. Visualization results and tables are exportable as png and csv files, respectively. The GUIs in (**A**,**B**) are provided as web applications. Multiple users can access these GUIs through equipped or external web browsers.

UNI-EM is a software collection for CNN-based EM image segmentation that includes ground truth generation, training, inference, postprocessing, proofreading, and visualization (Fig. 1). UNI-EM is written in Python 3.6 and runs on Microsoft Windows 10 (64 bit) and Linux. We also built UNI-EM on the Python application bundler called Pyinstaller on Windows 10; thus, users can employ UNI-EM without installing the Python programming environment. CPU and GPU versions are available, and users can maximize the performance using the GPU version if the computer is equipped with an NVIDIA GPU card that has a NVIDIA compute capability over 3.5. The developed Python source code with an online manual is available at the public repository GitHub (https://github.com/urakubo/UNI-EM).

The main component of UNI-EM is a web-based proofreading software, Dojo (Fig. 1A)^25^. Dojo provides a graphical user interface (GUI) for users to correct mis-segmentation arising from automated EM segmentation. We extended Dojo to have file import/export functions (png/tiff files), a more sophisticated GUI, and multiscale paint functions. With these extensions, users can employ Dojo not only for proofreading, but also for ground truth generation, both of which are important manual operation procedures for CNN-based segmentation. Dojo consists of a Python-based web/database server and an HTML5/JavaScript-based client interface. The server–client system allows multiple users to access it simultaneously through web browsers in an OS-independent manner. UNI-EM equips its own web browser called Chromium for the standalone use of Dojo with either a mouse or a stylus.

We also developed a new 3D annotator to visualize the proofread objects in a 3D space as well as to annotate these segmented objects (Fig. 1B). This annotator is a surface mesh-based 3D viewer with a table that shows segmented objects. Users can change the color and brightness of target objects and export the visualization results as png image files, as well as assign a name to each object and put marker points on the object surface. The results of these annotations can be exported as csv files for further analyses.

We then implemented a U-Net equipped with a GUI as a representative 2D CNN for EM-image segmentation^8^. U-Net has characteristic contracting and expansive convolution layers with skip connections, which showed the highest segmentation accuracy in the EM Segmentation Challenge in the International Symposium on Biomedical Imaging 2012 Conference (ISBI 2012) at the time of publication^8^. We similarly implemented ResNet^9^, Highway-Net^10^, and Dense-Net^11^. All of the CNNs accept single-channel (gray-scale) or three-channel (RGB) images. Users can choose any combination of these CNNs, loss functions, training times, and data augmentation methods, through a command panel.

We further wrapped FFNs as a representative algorithm of 3D CNN-based neuron segmentation^12^. FFNs are a recurrent CNN that infers a volume mask indicating whether target voxels belong to the centered object, and the inference program obtains an overall volume mask for each object using a flood filling algorithm. FFNs have outperformed many other algorithms in the segmentation accuracies of FIB-25^13^ and SNEMI3D^14^. Users can conduct a series of FFN processes, i.e., preprocessing, training, inference, and postprocessing, through a command panel.

The 2D CNNs and 3D FFNs were implemented on the Tensorflow framework^24^. Its resource monitor Tensorboard can be conveniently accessed from UNI-EM, so users can easily check the status of a target CNN, such as the network topology and loss function. UNI-EM also has a GUI for 2D/3D classic image filters. Users can apply multiple image filters simultaneously to a stack of 2D images in a single execution. The target images of the CNNs and classic filters are opened/closed through a folder manager. Further, users can implement new CNN models through the “Plugin” dropdown menu. Details on how to implement a new CNN are outlined in the online manual (see Data availability).

### Example workflows

In this section, we demonstrate how users can benefit from UNI-EM by introducing two example workflows. The first one is mitochondria segmentation using 2D CNNs, and the second one is neuron segmentation using 3D FFNs. In both cases, we targeted an EM image stack that was prepared for SNEMI3D^26^. The target brain region is the mouse somatosensory cortex, and the EM images were obtained using scanning electron microscopy (SEM) in combination with an automatic tape-collecting ultra-microtome system (ATUM/SEM)^14^. The spatial resolution of the EM images was 6 nm per pixel (xy-plane) and 30 nm per Z slice, and the overall image volume was 6.1 × 6.1 × 3 μm. The images were passed through a contrast-limited adaptive histogram equalization filter (CLAHE; block size 127, histogram bins 256, max slope 1.50) before segmentation.

#### Case 1: Mitochondria segmentation using 2D CNN

**Figure 2:**
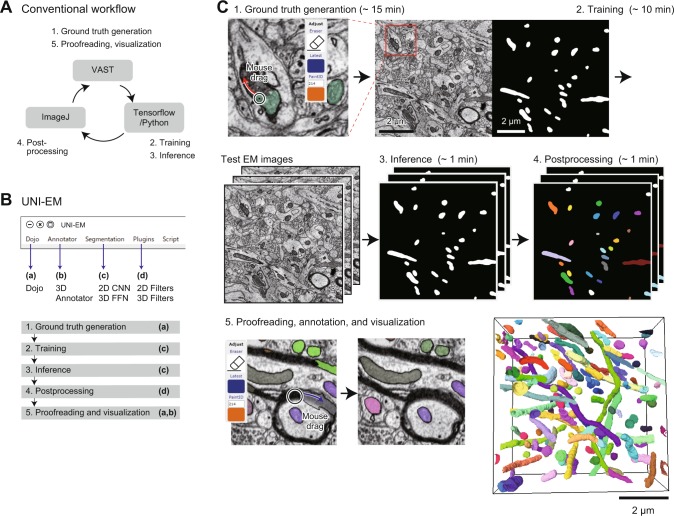
Example workflow 1: Mitochondria segmentation using 2D CNN. (**A**) Conventional workflow. Users first paint the regions of mitochondria of a target EM image using painting software, e.g., VAST lite (1, top)^22^. This mitochondrial segmentation image (ground truth) and the EM image are transferred to Tensorflow/Python for CNN training and inference (2,3; right). Inferred segmentation is then postprocessed (4, left), e.g., using imageJ, proofread and visualized by VAST lite (5, top). Such relays between software packages are necessary. (**B**) UNI-EM dropdown menu. A series of software (a-d) is located for the CNN-based segmentation (1–5). Standard png/tiff file format is used to connect these software packages. (**C**) Workflow in UNI-EM. Extended Dojo supports paint functions (1; top, left) to draw mitochondrial segmentation (top, right). Users can conduct CNN training (2) and inference (3) through a control panel. A labeling function is also implemented for postprocessing (4, each label is denoted by color). These segmented images are proofread by Dojo (5, left), and visualized by the 3D annotator (5, right).

Mitochondria are abundant where the metabolic demand is high, such as in synapses and active axons^27,28^, and their detection and quantification are important for treating neuronal diseases^29^. Because mitochondria possess characteristic oval shapes^30^, their segmentation is a good target for 2D CNN-based segmentation^31^. However, it is not accessible to inexperienced users (Fig. 2A). Firstly, inexperienced users need to learn how to use Python, install a CNN framework, and download an implementation of the target CNN from a public repository. The other software packages need to be installed for ground truth generation, post-processing, and proofreading (Fig. 2A). These steps can be learned, but a major hurdle is the transfer of data, especially to a CNN, when the users must convert EM/segmentation images into HDF5 or npz format files. To confirm that UNI-EM decreases the arduousness of these tasks (Fig. 2B), two test users (H.K. and Y.F.) who were not skilled in Python programming were requested to perform the following procedure (Fig. 2C):

1. Ground truth generation. The test users painted the mitochondrial regions of a single EM image using UNI-EM (Dojo). The generated ground truth was exported as an 8-bit grayscale PNG file (~20 min).
2. Training. A 16-layer ResNet with a least-square loss function was trained using the ground truth (~10 min computation time).
3. Inference. The trained ResNet was applied to test the EM images to obtain inferred 2D segmentation (~1 min).
4. Postprocessing. The inferred 2D segmentation images were binarized, and then each isolated region in 3D space was labeled with a specific ID number (~10 min).
5. Proofreading, annotation, and visualization. The test users proofread it with Dojo and visualized it with the 3D annotator (~30 min).

The test users successfully conducted the above procedure within the time indicated in parentheses and obtained the instance segmentation of mitochondria. The segmentation accuracy was sufficiently high without any proofreading (Fig. 2C, bottom and right panel; RAND score: 0.85; see Methods), as expected from published results on 2D CNN-based segmentation^31,32^. The detailed instructions for the mitochondria segmentation task can be found at the public repository GitHub (see Data availability).

**Figure 3:**
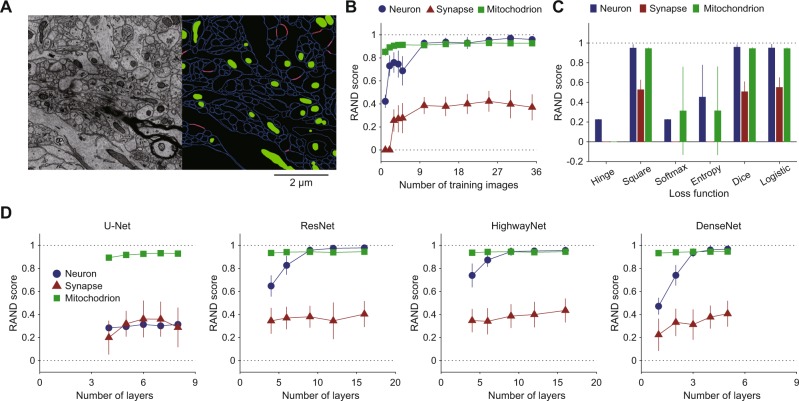
Performance survey in 2D CNN-based segmentation of neurons, synapses, and mitochondria. (**A**) One of target EM images (left, SNEMI3D) and ground truth segmentation (right). Each image panel has 1024 × 1024 voxels (3 nm/voxel in x-y plane), and 100 z-slices (3 nm/voxel in z slice). In the right panel, blue and red lines indicate cellular membranes and synapses, respectively, and green areas indicate mitochondria. (**B**) Training image number dependence of segmentation accuracy (n = 15, mean ± SD; RAND score, see Methods). The RAND score approaches 1 if the inferred segmentation is similar to the ground truth. (**C**) Loss function dependence of segmentation accuracy (n = 60, mean ± SD). Here, “Square” denotes least square, “Softmax” denotes SoftMax cross-entropy, and “Entropy” denotes multi-class and multi-label cross-entropy. (**D**) Network topology dependence of segmentation accuracy (n = 15, mean ± SD). In B-D, all of the parameters except the target parameters were set as follows: the number of training images: 1; loss function: least square; network topology: ResNet; number of layers: 9; number of training epochs: 2000; number of training images: 5 (standard CNN). The 2000 training epochs gave steady states of their losses.

In the above process, we requested the test users to use a 16-layer ResNet with a least-square loss function for mitochondrial segmentation. This request was determined based on the following quantitative survey on the segmentation of mitochondria, synapses, and neurons (Fig. 3A). Here we utilized the RAND score as a measure of segmentation accuracy (see Methods). The larger RAND score denotes higher accuracy. We first confirmed that only one ground truth image was sufficient for the segmentation of mitochondria (Fig. 3B), and 10 ground truth images were sufficient for neurons and synaptic segmentations. We then confirmed that the square, dice, and logistic loss functions were appropriate for segmentation (Fig. 3C). All of the 2D CNN types showed high accuracy in mitochondria segmentation (Fig. 3D, green lines; >0.9 RAND score). In addition, U-Net was not appropriate for membrane segmentation (Fig. 3D, red line; ~0.3 RAND score), and the segmentation accuracies in synapses are not high regardless of the type of CNN (Fig. 3D; ~0.3 RAND score). The accuracy of mitochondria segmentation in a standard CNN (network topology: ResNet; loss function: least square; number of layers: 9; training epochs: 2000; number of training images: 5) was indeed comparable with the accuracy in a recent 3D CNN-based, state-of-the-art algorithm^32^. The segmentation accuracy of the 3D CNN was quantified as Jaccard 0.92, Dice 0.96, and conformity 0.91 (semantic segmentation; ATUM/SEM data), whereas that of our standard 2D CNN was quantified as Jaccard 0.91, Dice 0.95, conformity 0.90 (semantic segmentation). Here, the larger scores of Jaccard, Dice, and conformity indicate higher accuracy^32^. Their 3D CNN requires 77 h of training time on a NVIDIA K40 GPU, whereas our standard CNN required only 5 min on a NIVDIA GTX1070 GPU. In addition, the 3D CNN was trained using the 3D ground truth, which requires excessive and tedious manual labeling. Overall, the implemented 2D CNN-based segmentations showed a sufficiently high and competitive accuracy compared to the current state-of-the-art mitochondrial segmentation algorithm^32^.

**Figure 4:**
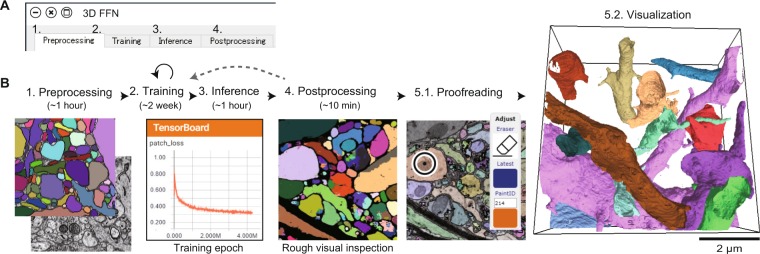
Example workflow 2: Neuron segmentation using 3D FFNs. (**A**) Control panel of 3D FFNs. Each tab (1–4) has one execute button for each FFN process. (**B**) Workflow. Computation times are indicated in parentheses. (1) Preprocessing. Ground truth segmentation and EM images are converted to intermediate files. (2) Training. FFNs are trained with the intermediate files. Users can monitor the progress of training using Tensorboard. (3) Inference. (4) Postprocessing. The program can also generate colored inferred segmentation for rough visual inspection. If the segmentation quality is insufficient, users can continue the training process. (5.1) Proofreading using Dojo. (5.2) Visualization by the 3D annotator.

#### Case 2: Neuron segmentation using 3D FFNs

We next asked a test user (N.Y.) to conduct neuron segmentation using 3D FFNs^12^, which is a primary topic in micro-connectomics. Various 2D and 3D CNNs have been proposed for accurate neuron segmentation^33,34^. FFNs currently show some of the highest segmentation accuracies in neuron segmentation^12^, although they require laborious work to generate the 3D ground truth. Users can generate the 3D ground truth using Dojo, but we recommend VAST lite for this purpose^22^. In the present case, we used the ground truth included in the SNEMI3D dataset. The test user successfully conducted the following procedure through the command panel (Fig. 4A):

1. Preprocessing. Stacks of target EM images and ground truth images were converted into FFN-specialized style files (~1 h computation time; Fig. 4B).
2. Training. FFNs were trained with the preprocessed EM-image/segmentation files (~2 weeks computation time on a NIVDIA GTX1080Ti GPU; Fig. 4B).
3. Inference. The trained FFNs were applied to a stack of test EM images for the inference of 3D segmentation (~1 h computation time on a NIVDIA GTX1080Ti GPU; Fig. 4B).
4. Postprocessing. The output segmentation files were converted into a PNG file stack (~10 min computation time; Fig. 4B).
5. Proofreading and visualization. The converted PNG files and EM images were imported into Dojo for proofreading as well as the 3D annotator for visualization (Fig. 4B).

**Figure 5:**
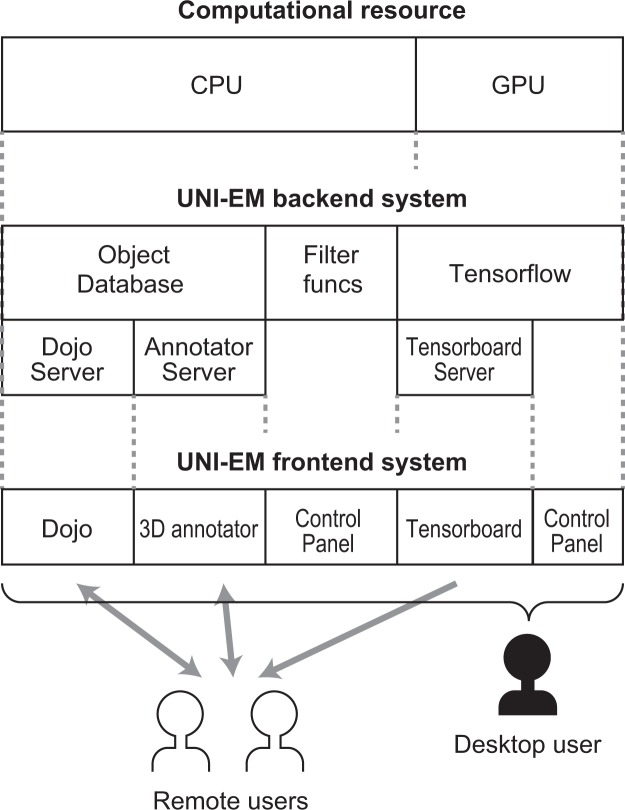
Underlying architecture of UNI-EM. UNI-EM has a heterogenous system. Present desktop computers have two types of computational resources: CPU and GPU (top). A GPU is used by Tensorflow for CNN computing (middle), which is not appropriate for shared use. Only the resource monitor Tensorboard can be used by remote users (bottom). Similarly, remote users can use proofreader Dojo and 3D annotator. Only a desktop user (silhouette person) can control all of the UNI-EM functions, including job submission for CNN computing such as training and inference.

Note that the trained FFNs directly inferred a 3D instance segmentation from a stack of 2D EM images. The FFNs gave a reasonably accurate neuron segmentation (Fig. 4B, right), whose RAND score was 0.84 (after 7 million training epochs; see Methods)^12^. This score was obtained without any postprocessing and specific parameter turning for the SNEMI3D dataset, and the topological structure of the neurites was well preserved in the segmentation results. Januszewski *et al*. reported a RAND score of 0.975 in the case of the SNEMI3D dataset^12^. This score was obtained with two additional processes: automated agglomeration of oversegmentation and a 2D watershed^12^. Thus, there is room for further improvement. Although FFNs require long training time (~2 weeks), users can benefit from their precise inference, which drastically decreases the subsequent proofreading work.

### System design

UNI-EM was developed under the Python development environment and Python bindings for v5 of the Qt application framework for GUI (PyQt5). The combination of Python and PyQt5 is typical for Python GUI desktop applications (e.g., Sommer *et al*.^19^), and UNI-EM utilizes this combination for GUI-equipped 2D CNNs and 3D FFNs (Fig. 5). The desktop application style is appropriate for CNN computing because CNN training/inference often occupies all of the GPU resources of a desktop computer, and the shared usage of a single GPU is ineffective. On the other hand, Dojo, the 3D annotator, and Tensorboard are web applications. The web application style provides remote accessibility to these applications; hence, multiple users can simultaneously use them (remote users in Fig. 5). Tensorboard enables the remote inspection of CNN training, Dojo enables multiple users to correct mis-segmentation simultaneously, and the 3D annotator enables multiuser annotation. Together, UNI-EM is comprised of desktop and web application systems, and this heterogeneity enables a wide range of applications from individual to shared use.

## Discussion

We presented a software package called UNI-EM for CNN-based automated EM segmentation. UNI-EM unifies pieces of software for CNN-based segmentation. We validated its effectiveness using two example workflows: mitochondria segmentation using a 2D CNN and neuron segmentation using 3D FFNs. Test users who did not possess Python programming skills were able to perform the overall procedure successfully, and the resulting segmentation accuracies were comparable to those of state-of-the-art methods. Therefore, UNI-EM is a beneficial tool for researchers with limited programming skills.

In recent years, the popularity of CNNs in generic image segmentation as well as EM image segmentation has greatly increased^7^. Numerous CNN-based segmentation algorithms have been proposed, and their source codes are often released along with journal publication. However, it is difficult to use such CNN source code as doing so often requires knowledge of Python and a CNN framework. In such situations, UNI-EM provides an opportunity for researchers to examine the effectiveness of multiple CNNs based on their own EM images, without knowledge of Python. Based on the results, they can decide if they want to use these CNNs professionally for large-scale segmentation. UNI-EM therefore functions as a testing platform.

Two-dimensional CNN-based segmentation combined with subsequent Z-slice connection into 3D objects is effective if the target objects have simple shapes like that of mitochondria. In the example workflow, the test users successfully extracted the oval-shaped mitochondria within 2 h, and the segmentation accuracy was higher than those of conventional machine learning methods such as AdaBoost^15^. The proposed approach is also effective for neuron segmentation if the users can utilize high-performance Z slice connectors, such as rule-based connectors^15^, multicut algorithms^35^, and the graph-based active learning of agglomeration^36^. Incorporation of these connectors into UNI-EM is an important future direction because the current UNI-EM only provides 3D labeling and 3D watersheds to connect the 2D segments.

Many 3D CNNs have been proposed for highly accurate neuron segmentation^12,34,37,38^. FFNs are one such 3D CNNs^12^, but we have to acknowledge two remaining barriers from its common use. First, FFNs require a long training period over one week. Second, they require a certain amount of 3D ground truth segmentation. In our experience, two-week labor was required to manually draw 3D ground truth using a sophisticated paint tool^22^. FFNs are of course still an excellent selection if we consider the time for manual correction of mis-segmentation arising from other segmentation methods.

The proofreading software Dojo with extensions is one of the main components of UNI-EM^25^. Similar to Dojo, numerous excellent proofreading and manual segmentation tools are available, e.g., Reconstruct^18^, Ilastik^19^, TrakEM2^39^, VAST lite^22^, Knossos^20^, webKnossos^40^, Microscopy Image Browser^21^, CATMAID^41^, NeuTu^42^, and Neuroglancer^43^. The primary advantage of Dojo is its web application architecture. A web application has numerous advantages; there is no need for the end users to install any software except for the web browser, OS independency, and cloud resource accessibility, and multiuser access is typically included. However, a distinct web/database server needs to be launched. To avoid this task, UNI-EM itself contains the backend web/database server of Dojo. Users can employ UNI-EM as both single-user and collaborative applications, without launching any distinct servers.

Almost all of UNI-EM programs are written in high-level interpreter languages, i.e., Python, JavaScript, HTML, and CSS, and only the matching cube mesh generator is currently written in a C++ compiler language. The interpreter languages generally have lesser abilities to manage CPU and memory resources and show reduced performance. On the other hand, CNN frameworks such as TensorFlow and PyTorch provide application programming interfaces on high-level languages, such as Python. Thus, users can easily incorporate new CNN models into UNI-EM. The instructions for extending UNI-EM are provided in an online manual (see Data availability).

## Methods

### RAND score

We utilized the foreground-restricted RAND score as a metric of segmentation performance^7^. The RAND score is defined as follows. Suppose *p*_*ij*_ is the joint probability that a target pixel belongs to object *i* of inferred segmentation and object *j* of ground truth segmentation (Σ_*ij*_ *p*_*ij*_ = 1). Subsequently, *s*_*i*_ = Σ_*j*_ *p*_*ij*_ is the marginal probability for the inferred segmentation, and *t*_*j*_ = Σ_*i*_ *p*_*ij*_ is the marginal probability for the ground truth segmentation. Subsequently, the RAND score, ${V}_{\alpha }^{{\rm{Rand}}}$, can be defined as follows:

$${V}_{\alpha }^{{\rm{Rand}}}=\frac{{\sum }_{ij}{p}_{ij}^{2}}{\alpha {\sum }_{k}{s}_{k}^{2}+(1-\alpha ){\sum }_{k}{t}_{k}^{2}},$$

where the RAND F-score *α* is set to be 0.5. The split score (*α* → 0) can be interpreted as the precision in the classification of pixel pairs as belonging to the same (positive class) or different objects (negative class). The merge score (*α* → 1) can be interpreted as recall. Generally, ${V}_{\alpha }^{{\rm{Rand}}}$ becomes equal to 1 if the segmentation is accurate. Note that, as utilized in a neuron segmentation contest^7^, the RAND scores of instance segmentation were obtained in the case of neuron segmentation in the 2D CNNs and FFNs (Figs. 2 and 4), i.e., isolated neurons were counted as independent objects. On the other hand, the RAND scores of semantic segmentation were obtained in the cases of synapses and mitochondria in the 2D CNNs (Fig. 2) to compare the scores with those in a 3D CNN^32^.

## Data Availability

The datasets generated and/or analyzed during the current study are available from the corresponding author upon reasonable request. UNI-EM is available at https://github.com/urakubo/UNI-EM.
